# Unraveling the link between the mistreatment of women during childbirth and postpartum depression: a prospective longitudinal study in Ethiopia and Guinea

**DOI:** 10.1016/j.eclinm.2025.103702

**Published:** 2025-12-18

**Authors:** Anteneh Asefa, Lenka Beňová, Bruno Marchal, Charlotte Hanlon, Tamba Mina Millimouno, Mariamawit Asfaw, Özge Tunçalp, Tom Smekens, Alexandre Delamou, Samson Gebremedhin

**Affiliations:** a Department of Public Health, Institute of Tropical Medicine, Antwerp, Belgium; b Department of Epidemiology and Population Health, London School of Hygiene and Tropical Medicine, London, UK; c Division of Psychiatry, Centre for Clinical Brain Sciences, University of Edinburgh, Edinburgh, Scotland, UK; d Department of Psychiatry, School of Medicine, College of Health Sciences, Addis Ababa University, Addis Ababa, Ethiopia; e Center for Innovative Drug Development and Therapeutic Trials for Africa (CDT-Africa), College of Health Sciences, Addis Ababa University, Ethiopia; f Centre d’Excellence d’Afrique Pour la Prévention et le Contrôle des Maladies Transmissibles (CEA-PCMT), Université Gamal Abdel Nasser de Conakry, Conakry, Guinea; g Centre National de Formation et de Recherche en Santé Rurale de Maferinyah, Forecariah, Guinea; h Department of Gerontology, Faculty of Medicine and Pharmacy, Vrije Universiteit Brussel (VUB), Brussels, Belgium; i Maternal, Child and Adolescent Health Service Lead Executive Office, Ministry of Health, Addis Ababa, Ethiopia; j School of Public Health, Addis Ababa University, Addis Ababa, Ethiopia

**Keywords:** Mistreatment, Postpartum depression, Perinatal mental health, Ethiopia, Guinea

## Abstract

**Background:**

The mistreatment of women during facility-based childbirth is widespread in sub-Saharan Africa and has a negative impact on women's mental health. We aimed to examine the association between childbirth-related mistreatment and postpartum depression in Ethiopia and Guinea.

**Methods:**

Between May 2023 and February 2024, we conducted a prospective longitudinal survey of pregnant women recruited from 22 health facilities in Addis Ababa, Ethiopia, and 20 health facilities in Conakry, Guinea. Participants were surveyed during the third trimester in health facilities and were followed up in the community for a second survey, which was conducted between 6 and 16 weeks postpartum. Depression was assessed using the Edinburgh Postnatal Depression Scale (EPDS), and mistreatment was measured across seven categories. We used multilevel mixed effects Poisson regression to assess the association between the number of mistreatment categories experienced and women's postpartum depression scores.

**Findings:**

Of the 859 women enrolled during pregnancy, 711 women completed the postpartum survey. 87.4% of women in Addis Ababa and 71.2% in Conakry had experienced at least one category of mistreatment. Symptoms suggestive of postpartum depression (EPDS ≥11) were reported by 20.9% of women in Addis Ababa and 31.0% in Conakry. After adjusting for antepartum depression, intimate partner violence, and other sociodemographic, obstetric, and health service-related characteristics, experience of each additional mistreatment category was associated with a 5% increase in women's postpartum depression scores (adjusted incidence rate ratio [AIRR] = 1.05, 95% CI: 1.01–1.09). Among women without symptoms suggestive of antepartum depression (EPDS <11), the effect was even greater, with an 11% increase in postpartum depression scores (AIRR = 1.11; 95% CI: 1.06–1.17).

**Interpretation:**

The strong association between mistreatment and postpartum depression, particularly among women without antenatal depressive symptoms, highlights the potential causal role of mistreatment and underscores the urgent need for coordinated, evidence-informed, and context-appropriate strategies to promote respectful maternity care and safeguard women's mental health.

**Funding:**

10.13039/501100003130Research Foundation Flanders (FWO file number 1261923N) and the 10.13039/100018370Institute of Tropical Medicine (ITM), Antwerp, Belgium, with support from the Flemish Government Department of Economy, Science and Innovation (EWI) and the Belgian Federal Directorate-General for Development Cooperation and Humanitarian Aid (DGD).

**Research in context**

**Evidence before this study**

There is an increasing body of evidence on the link between mistreatment during childbirth and postpartum depression. A multi-country retrospective study in Ghana, Guinea, Myanmar and Nigeria found that women who experienced physical or verbal abuse, or stigma and discrimination, had a 19%–69% higher risk of postpartum depression than those who did not.

**Added value of this study**

Using a prospective longitudinal design and accounting for antenatal depression, relational factors (experience of intimate partner violence and social support), and key sociodemographic, obstetric, and service-related characteristics, this study provides a more precise estimate of the association between mistreatment during childbirth and postpartum depression scores.

**Implications of all the available evidence**

Given the complexity of both mistreatment and postpartum depression, and their intersecting contributors, a multidimensional and systems-oriented approach is needed to inform the development, implementation, and scalability of effective interventions. Approaches that prioritise trust-building and the participatory engagement of women and health workers are essential. Further research is required to understand the organisational, social, and health system-related factors that contribute to mistreatment during childbirth in health facilities.

## Introduction

Remarkable progress has been made in improving access to lifesaving maternal and newborn health services in low- and middle-income countries (LMICs). In sub-Saharan Africa (SSA)—where 70% of global maternal deaths and 46% of global neonatal deaths occurred in 2020—maternal mortality ratio and neonatal mortality rate fell by 33% and 37%, respectively, between 2000 and 2020.^1^^,^^2^ However, poor quality and lack of woman-centred maternity care remain critical barriers to the provision and uptake of lifesaving maternal and newborn health interventions in the region.^1^^,^^3^^,^^4^ These challenges contribute to preventable maternal and newborn deaths and hinder progress toward achieving the ambitious Sustainable Development Goals (SDGs) targets of reducing maternal and newborn deaths.^5^

Modelled estimates from 81 LMICs indicate that improving the quality of maternal and newborn health care could reduce maternal and newborn deaths by 28%.^6^ A particularly pervasive form of poor care quality is the mistreatment of women during childbirth in health facilities—often referred to as obstetric violence or disrespect and abuse.^7^ Mistreatment can manifest in diverse interpersonal and structural forms, including verbal abuse, physical abuse, non-consented care, lack of privacy and confidentiality, detention in health facilities, neglect, discrimination, and inhumane and substandard care.^8^

Mistreatment of women has been widely reported globally and is most prevalent in resource-limited settings.^8^ A systematic review of 33 studies from SSA reported a pooled prevalence of mistreatment during childbirth of 44%.^9^ A recent systematic review from Ethiopia reported a pooled prevalence of mistreatment of 49%, whereas studies from Addis Ababa reported a prevalence ranging from 70% to 78%.^10^, ^11^, ^12^ In Guinea, a community-based survey of postpartum women in urban areas found that mistreatment was widespread, with the most common forms being non-consented use of episiotomy (73%), lack of privacy during vaginal examinations (50%), verbal abuse (27%), and physical abuse (19%).^13^

The mistreatment of women during childbirth not only violates their right to high-quality, person-centred care, but also affects their physical, psychological, and emotional well-being. It also undermines their trust in health systems and discourages them from seeking future care.^14^, ^15^, ^16^ Regarding psychological sequalae, there are increasing reports of experience of postpartum depression among women who have been mistreated during childbirth. A multi-country retrospective study in Ghana, Guinea, Myanmar and Nigeria found that women who experienced physical or verbal abuse, or stigma and discrimination, had a 19%–69% increased risk of postpartum depression compared to those who did not.^17^

The objective of this study was to prospectively examine the association between the mistreatment of women during facility-based childbirth and postpartum depression in Ethiopia and Guinea, accounting for the effects of antepartum depression and women's relational characteristics.

## Methods

### Study design

This longitudinal quantitative study is part of a larger mixed-methods study conducted in Addis Ababa, Ethiopia, and Conakry, Guinea.^18^ The broader study aims to understand the relationship between the mistreatment of women during childbirth in health facilities and postpartum depression, and to explore the capacity of health systems to provide integrated perinatal mental health services.^18^ We conducted a prospective longitudinal survey, recruiting women who sought antenatal care in selected health facilities in the two cities in the third trimester (28–41 weeks gestation) and followed them up to 16 weeks postpartum. Women were assessed twice: first, upon their exit from facility-based antenatal care visits during the third trimester of pregnancy (facility-based survey at time of recruitment), and second, six to 16 weeks after childbirth (community-based survey).

### Sample size, sampling, and recruitment

The minimum sample size required was 434 for Ethiopia and 408 for Guinea. We calculated the sample size using the assumptions for calculating the difference between two dependent means, accounting for a power of 0.8, a significance level of 0.05, a design effect of 1.5, an effect size of 0.2, and an estimated dropout rate of 15% between the two surveys. Additionally, we increased the sample size by 21% for Ethiopia and 16% for Guinea to account for the proportion of women giving birth at home in urban areas and therefore not exposed to potential mistreatment in health facilities.

Women were recruited between May 2023 and February 2024 from antenatal clinics in 22 health facilities in Addis Ababa (18 public and 4 private) and 20 health facilities in Conakry (16 public and 4 private) using a stratified cluster sampling method. Facilities were first stratified by ownership (public or private), and then by the level of care they provide (hospitals or health centres or health posts/clinics), as indicated in the protocol.^18^ Women were enrolled consecutively until the target sample size for each facility was achieved (20 in most cases). Eligible women—those in the third trimester of pregnancy and living in the respective city for at least six months prior to the study—were invited to participate in the survey at the end of their antenatal care visits. Women who agreed to participate in the first survey round were informed that they would also receive an invitation to participate in the second round during the postpartum period. During the first survey round, participants’ names, their unique four-digit participant codes, and their telephone numbers were recorded on separate, password-protected tracking sheets. These sheets were used to contact participants for the second survey round and to link data from the two rounds using the unique participant codes. The tracking sheets were securely stored by the local research coordinators and no data from the questionnaire were linked to them.

We recruited 859 women (442 in Ethiopia and 417 in Guinea) for the first survey round. A total of 711 women (373 in Ethiopia and 338 in Guinea) participated in both rounds, representing a loss to follow-up of 15.6% in Ethiopia and 18.9% in Guinea. Among those who participated in both rounds, 688 women (372 in Ethiopia and 316 in Guinea) gave birth either in a health facility, on the way to a health facility, or at home but subsequently sought care at a health facility, and were included in the analysis.

### Variables

#### Dependent variable

The dependent variable in this study is postpartum depression score measured using the Edinburgh Postnatal Depression Scale (EPDS), validated for use in Ethiopian^19^^,^^20^ and West African settings.^21^ The EPDS comprises 10 items, each scored from 0 to 3, resulting in a minimum possible score of 0 and a maximum of 30. A recent systematic review and meta-analysis of 58 studies evaluated the accuracy of the EPDS in detecting postpartum depression, suggesting that a cut-off score of 11 or higher provides better combined sensitivity and specificity.^22^ We used this cut-off value to describe women with symptoms suggestive of postpartum depression but treated the EPDS score as a continuous variable in the regression model to quantify how the scores varied based on women's experiences of mistreatment.

#### Independent variables

The independent variable was mistreatment during facility-based childbirth, a continuous variable counting the number of categories of mistreatment experienced (ranging from 0 to 7; verbal abuse, physical abuse, non-consented care, lack of information, privacy and confidentiality, neglect and discrimination, refusal of preference, and detention in health facilities). We used a 25-item questionnaire with yes or no response options, grouped under the seven categories, to assess mistreatment during facility-based childbirth and immediately afterwards (Supplementary File S1). The tool was adapted from previously validated tools used in Ethiopia^10^^,^^23^ and Ghana, Guinea, Myanmar, and Nigeria.^13^^,^^24^

##### Sociodemographic variables

Woman's age (in completed years), highest educational status, and worry about feeding the family during the four weeks preceding the second survey round.

##### Obstetric and service related characteristics

Parity at the time of the first survey, complications during the index pregnancy, outcome of the index pregnancy, place of childbirth, ownership of the childbirth facility (public or private), referral status to a health facility, mode of childbirth, complication during childbirth, procedure for an assisted delivery, postnatal care receipt during the postpartum period, duration (in days) between date of childbirth and date of second round survey, and gender of the main health worker who assisted the woman during childbirth and/or immediate postpartum care.

##### Relational characteristics

Social support score (out of 30) and experience of spousal violence. Social support was measured using the Maternity Social Support Scale, which consists of six items, each scored on a five-point scale (0–5), resulting in a possible total score ranging from 0 to 30.^25^ Experience of at least one of the three forms of spousal violence (emotional, physical, and sexual) in the 12 months prior to the survey (for the survey during pregnancy) and since childbirth (for the postpartum survey) were assessed using a 12-item standardised questionnaire with ‘yes’ or ‘no’ options designed by the DHS Program.^26^

### Data collection

Data were collected using a structured questionnaire (Supplementary File S1) by 11 trained female data collectors (5 in Ethiopia and 6 in Guinea)—doctors, nurses, and clinical officers with advanced training in public health—who were not affiliated with the selected health facilities. The questionnaire was originally prepared in English, then translated into Amharic (for Ethiopia) and French (for Guinea), and administered in Amharic in Ethiopia and in Maninka, Pular, and Susu languages in Guinea. Data collectors received intensive training (one session per country for each round of the surveys) on survey administration organised by the principal investigator (AA) and country co-investigators (SG in Ethiopia and AD and TMM in Guinea). Given the sensitivity of the study topics—intimate partner violence, mistreatment during childbirth, and perinatal depression—the training included practical sessions on psychosocial support and the referral of women for further counselling and support. The tools were pretested in one health facility in each city, after which adjustments were made to the structure and organisation of the questionnaire. The survey questionnaire was administered face-to-face at health facilities for the survey during pregnancy and at the participants’ homes or preferred locations for the postpartum survey. So, no mistreatment related questions were asked at health facilities. Data were collected electronically using the KoboToolbox toolkit, checked and approved daily by the local field supervisors, and uploaded to the server managed by the principal investigator.

### Data management and analysis

Data from the two survey rounds were cleaned and merged on a country-by-country basis, and then the merged datasets from the two countries were appended for analysis using StataSE V.16 (StataCorp, College Station, Texas, United States). We present descriptive statistics of variables by country and the results of the regression models analysing the association between these variables and postpartum depression score using the combined dataset of both countries.

We calculated variance inflation factors (VIF) for the independent variables to check for multicollinearity, including between the seven mistreatment categories. All VIF values were below 1.3, well under the conservative cut-off of 3, indicating no collinearity concerns between the mistreatment categories. A multilevel mixed effects Poisson regression analysis was conducted to identify the association between the number of mistreatment categories experienced and postpartum depression score variables while adjusting for possible confounders. Poisson regression was chosen over linear regression because the distribution of the EPDS score variable resembled an exponential distribution much more than a normal distribution. This violates the homoscedasticity assumption of linear models, making Poisson regression a more appropriate choice.

In this study, we constructed and compared three models: a null model (intercept only) with the intercept as a fixed effect and random effects for country and health facilities (Model I); a model including the number of mistreatment categories experienced as a fixed effect, along with random effects for country health facility (Model II); and a model including the number of mistreatment categories experienced, as well as potential confounders including sociodemographic, obstetric and service-related, and relational characteristics as fixed effects,^13^^,^^23^^,^^27^ with random effects for country and health facility (Model III). Given the strong evidence on the link between antepartum depression and postpartum depression^28^ and interactions between antepartum depression and the various categories of mistreatment,^27^ we included an interaction term between antepartum depression score and the number of mistreatment categories experienced to capture their combined effects on postpartum depression score in Model III. Additionally, due to existing evidence on the increased effect of mistreatment on postpartum depression among women without symptoms suggestive of antepartum depression (EPDS <11), we conducted a subgroup analysis for model III (Supplementary File S2). We included country and health facility as random effects in all three models to account for the potential lack of independence among women living in the same country and receiving antenatal care at the same health facility. Observations (n = 25) with missing values for the variables included in the regression models were excluded. We conducted a likelihood ratio test to select the better-fitting model from the three models. Adjusted incidence rate ratios (AIRRs) with their corresponding 95% confidence intervals (CIs) were used to assess the association between the independent variables and the dependent variable. To see the association between the individual categories of mistreatment and postpartum depression, we also ran Model III to calculate the effect size (AIRR) for each category of mistreatment (Supplementary File S3).

### Ethics

The study was approved by the Institutional Review Board of the Institute of Tropical Medicine, Antwerp (reference number: 1672/23), the Ethics Committee of Addis Ababa City Administration Health Bureau (reference number: A/A/H/10932/229), and the Comité National d'Éthique Pour la Recherche en Santé en Guinée (reference number: 095/CNERS/23). Written informed consent was obtained from all study participants prior to participation. For illiterate women, data collectors read the information sheet out loud and confirmed their willingness to participate and signed the consent form on their behalf in the presence of a witness. Letters of permission and support were obtained from Addis Ababa City Administration Health Bureau, Conakry City Health Directorate, and the health facilities involved in the study. Data collection adhered to the ethical principles outlined in the Declaration of Helsinki and the European Union's General Data Protection Regulation (GDPR).

### Role of the funding source

The funding bodies had no role in the study design, data collection, data analysis, data interpretation, the writing of the report, or the decision to submit this paper for publication.

## Results

### Participants’ characteristics during pregnancy

The median (IQR) age of participants at the time of the first survey during pregnancy was 28 (25–31) and 25 (21–29) years in Ethiopia and Guinea, respectively. The majority (97.0% in Ethiopia and 91.1% in Guinea) of women were in marital union. There were significant differences between participants in the two countries in terms of educational status, pregnancy intention, parity, experience of complication(s) during pregnancy, and antepartum depression score (Table 1). More than one-third of participants (34.5%) in Guinea reported experiencing at least one form of intimate partner violence in the 12 months preceding the survey, compared to 30.1% of participants in Ethiopia. The median (IQR) antepartum depression score was higher in Guinea 8 (5–13) compared to Ethiopia 5 (2–8).

**Table 1:** Key sociodemographic, obstetric, and relational characteristics of participants during pregnancy.

Characteristics	Ethiopia (n = 372)	Guinea (n = 316)	P value
Age group at time of survey (years)			
15–24	76 (20.4%)	151 (47.8%)	<0.001
25–34	257 (69.1%)	140 (44.3%)	
35–44	39 (10.5%)	25 (7.9%)	
Median (IQR)	28 (25–31)	25 (21–29)	
Marital status at time of survey			
Not in union (single, separated or divorced)	11 (3.0%)	28 (8.9%)	0.001
In union (married or cohabiting)	355 (97.0%)	288 (91.1%)	
Educational status			
No education	20 (5.4%)	83 (26.3%)	<0.001
Primary school	96 (25.8%)	101 (31.9%)	
Completed primary school	34 (9.1%)	30 (9.5%)	
Secondary school	71 (19.1%)	37 (11.7%)	
Completed secondary school	31 (8.3%)	17 (5.4%)	
Higher education	120 (32.3%)	48 (15.2%)	
Pregnancy intention of current pregnancy			
Intended	280 (75.3%)	211 (66.8%)	0.014
Mistimed or unwanted	92 (24.7%)	105 (33.2%)	
Parity at time of the survey during pregnancy			
Nullipara (no previous births)	135 (36.3%)	89 (28.2%)	0.023
Primipara or Multipara (1 or more previous births)	237 (63.7%)	227 (71.8%)	
Complication/s during current pregnancy			
No	302 (81.2%)	283 (89.8%)	0.001
Yes	70 (18.8%)	32 (10.2%)	
Missing	–	1	
Experienced any forms of IPV in the 12 months preceding the first survey during pregnancy			
No	223 (59.9%)	148 (46.8%)	<0.001
Yes	112 (30.1%)	109 (34.5%)	
Prefer not to say	26 (7.0%)	31 (9.8%)	
Was not in union	11 (3.0%)	28 (8.7%)	
Antepartum depression			
Antepartum depression score (max 30): Median (IQR)	5 (2–8)	8 (5–13)	<0.001
Symptoms suggestive of antepartum depression (EPDS ≥11)	62 (16.9%)	111 (35.1%)	<0.001
Missing	4	–	

### Participants’ birth-related and postpartum characteristics

The mean duration between childbirth and the second survey round in postpartum was 8.3 weeks in Ethiopia and 7.7 weeks in Guinea (Table 2). Participants' average social support score out of 30 was higher in Ethiopia (22.4) than in Guinea (22.0). Just over half of participants in Ethiopia had a postnatal checkup by a health worker before discharge from a health facility or at home (51.9%), compared to 46.2% in Guinea. Nearly half (45.9%) of the participants in Guinea reported to have worried about feeding their family in the two weeks prior to the postpartum survey, compared to 30.3% in Ethiopia. Regarding participants’ service-related characteristics, 83.3% of participants in Ethiopia and 66.6% in Guinea gave birth or received immediate postnatal care in a public facility. Additionally, 28.8% of participants in Ethiopia were admitted to the facility where they gave birth or received immediate postnatal care upon referral from another facility, compared to only 13.0% in Guinea. There was a significant difference in mode of childbirth between participants in the two countries, with 38.3% of births being Caesarean in Ethiopia compared to 10.2% in Guinea. About a fifth (20.7%) of the participants in Ethiopia reported having had complications during childbirth (17.5% in Guinea) and 29.3% had experienced at least one of the three forms of IPV since childbirth (18.0% in Guinea).

**Table 2:** Key obstetric, relational, and service-related characteristics of participants: based on the postpartum survey.

Characteristics	Ethiopia (n = 372)	Guinea (n = 316)	P value
Number of weeks between childbirth and postpartum survey			
Mean ± SD	8.3 ± 2.0	7.7 ± 2.4	<0.001
Social support score at time of postpartum survey			
Mean ± SD	22.4 ± 5.6	22.0 ± 6.4	0.005
Had postnatal checkup after childbirth			
No	179 (48.1%)	170 (53.8%)	0.138
Yes	193 (51.9%)	146 (46.2%)	
Pregnancy outcome			
Live birth	366 (98.4%)	296 (93.7%)	0.001
Stillbirth or early neonatal loss	6 (1.6%)	20 (6.3%)	
Worried about feeding family in two weeks preceding the survey			
No	258 (69.7%)	171 (54.1%)	<0.001
Yes	112 (30.3%)	145 (45.9%)	
Missing	2	–	
Ownership of facility of childbirth/immediate postnatal care			
Public	62 (16.7%)	105 (33.4%)	<0.001
Private	310 (83.3%)	209 (66.6%)	
Missing	1	1	
Had referral to the facility of childbirth/immediate postnatal care			
Yes	107 (28.8%)	41 (13.0%)	<0.001
No	265 (71.2%)	275 (87.0%)	
Gender of the main health worker who assisted the woman during childbirth/immediate postpartum care			
Female	159 (42.7%)	255 (80.7%)	<0.001
Male	212 (57.0%)	61 (19.3%)	
Missing	1	–	
Mode of childbirth			
Vaginal	227 (61.7%)	281 (89.8%)	<0.001
Caesarean	141 (38.3%)	32 (10.2%)	
Missing	4	3	
Had an assisted vaginal birth			
No	299 (81.3%)	236 (75.6%)	0.075
Yes	69 (18.7%)	76 (24.4%)	
Missing	4	4	
Complication(s) during childbirth			
No	294 (79.3%)	260 (82.5%)	0.275
Yes	77 (20.7%)	55 (17.5%)	
Missing	1	1	
Experienced any form of IPV between childbirth and postpartum survey			
No	244 (65.6%)	216 (68.4%)	<0.001
Yes	109 (29.3%)	57 (18.0%)	
Prefer not to say	5 (1.3%)	5 (1.6%)	
Was not in union	14 (3.8)	38 (12.0%)	

### Experience of mistreatment during childbirth

The postpartum survey showed that more than four-fifths (87.4%) of the participants in Ethiopia experienced at least one of the seven forms of mistreatment, compared to 71.2% in Guinea (Table 3). Among those who reported experiencing at least one form of mistreatment, the mean number of mistreatment categories experienced was 2.9 in Ethiopia and 1.9 in Guinea. Across all seven categories of mistreatment, prevalence was higher in Ethiopia, with significant differences between the two countries, except for detention in a health facility, which was comparable at around 5%. In Ethiopia, the most common form of mistreatment category was “Lack of information, privacy, and confidentiality” (65.9%), whereas in Guinea, it was “Refusal of preference” (37.5%). About a fifth of participants in Ethiopia experienced “Physical abuse” (23.0%) or “Verbal abuse” (18.0%), compared to 4.8% and 7.3% in Guinea. “Neglect or discrimination” was experienced by a third (33.6%) of participants in Ethiopia, compared to 18.4% in Guinea (Table 3).

**Table 3:** Experience of mistreatment during childbirth by categories and postpartum depression.

Characteristics	Ethiopia	Guinea	P value
Mistreatment categories experienced	(n = 372)	(n = 316)	
Lack of information, privacy, and confidentiality	245 (65.9%)	111 (35.1%)	<0.001
Non-consented care	231 (62.1%)	109 (34.5%)	<0.001
Refusal of preference	165 (57.7%)	99 (37.5%)	<0.001
Neglect and discrimination	125 (33.6%)	58 (18.4%)	<0.001
Physical abuse	89 (23.0%)	15 (4.8%)	<0.001
Verbal abuse	67 (18.0%)	23 (7.3%)	<0.001
Detention in health facility	22 (5.9%)	15 (4.8%)	0.494
Any of the seven categories	325 (87.4%)	225 (71.2%)	<0.001
Number of mistreatment categories experienced: Mean ± SD	2.9 ± 1.5	1.9 ± 1.1	<0.001
Postpartum depression	(n = 367)	(n = 316)	
Postpartum EPDS score: Median (IQR)	5 (1–9)	7 (4–12)	<0.001
Symptoms suggestive of postpartum depression (EPDS ≥11)	77 (20.9%)	98 (31.0%)	0.003

### Postpartum depression and its association with experience of mistreatment during childbirth

The median (IQR) postpartum depression score of participants was 5 (1–9) in Ethiopia and 7 (4–12) in Guinea (p < 0.001) (Table 3). There was a statistically significant difference in the prevalence of symptoms suggestive of postpartum depression—using an EPDS score of 11 or higher as a cut-off—between participants from the two countries (31.0% in Guinea and 20.9% in Ethiopia; p = 0.003).

As shown in Table 4, the intraclass correlation coefficient (ICC) from Model I indicates that 17.5% of the variation in women's postpartum depression scores can be attributed to differences between health facilities within countries. Model II, the model with the number of mistreatment categories experienced, had a better fit than Model I and showed that 19.1% of the variation in women's postpartum depression scores can be attributed to differences between countries and health facilities. The model that included the number of mistreatment categories and other independent variables as fixed effects, and random effects for country and health facility (Model III), was the best fitting model (p < 0.001). Accordingly, we report the results of the analyses from Model III, focussing on both random effects (measures of variation) and fixed effects (measures of association) for women's postpartum depression scores.

**Table 4:** Multilevel mixed-effects model assessing the association between mistreatment and postpartum depression scores.

Variables	Model I (n = 683)	Model II (n = 683)	Model III (n = 663)^a^	
	AIRR (95% CI)	AIRR (95% CI)	AIRR (95% CI)	p value
(A) Fixed effects				
(Intercept)	5.80 (3.65–9.23)	4.73 (2.78–8.05)	6.83 (3.94–11.86)	
Number of mistreatment categories experienced		1.09 (1.07–1.11)	1.05 (1.01–1.09)	0.011
Antepartum depression score			1.04 (1.03–1.05)	<0.001
Number of mistreatment categories experienced^a^ APD score			1.00 (0.99–1.01)	0.086
Age (in years) at the time of the survey during pregnancy			1.01 (1.002–1.02)	0.011
Parity during pregnancy				
Nullipara (no previous births)			Ref.	
Primipara or Multipara (1 or more previous births)			0.95 (0.88–1.04)	0.291
Complication(s) during pregnancy				
No			Ref.	
Yes			1.07 (0.99–1.16)	0.074
Experienced any form of IPV in the 12 months preceding the survey during pregnancy				
No			Ref.	
Yes			1.03 (0.96–1.11)	0.420
Prefer not to say			0.96 (0.84–1.10)	0.588
Was not in union			0.86 (0.71–1.05)	0.140
Social support score during the postpartum survey			0.95 (0.94–0.96)	<0.001
Pregnancy outcome				
Live birth			Ref.	
Stillbirth or early neonatal loss			1.38 (1.21–1.58)	<0.001
Had postnatal checkup after childbirth				
No			Ref.	
Yes			0.89 (0.82–0.95)	<0.001
Worried about feeding family in the two weeks preceding the postpartum survey				
No			Ref.	
Yes			1.46 (1.36–1.56)	<0.001
Mode of childbirth				
Vaginal			Ref.	
Caesarean			1.26 (1.14–1.39)	0.118
Had procedure for assisted vaginal delivery				
No			Ref.	
Yes			1.15 (1.06–1.25)	0.001
Complication/s during childbirth				
No			Ref.	
Yes			1.07 (0.99–1.16)	0.001
Experienced any form of IPV between childbirth and postpartum survey				
No			Ref.	
Yes			1.10 (1.02–1.19)	0.020
Prefer not to say			0.86 (0.66–1.12)	0.262
Was not in union			0.70 (0.56–0.87)	0.001
(B) Random effects				
Country				
Variance	0.09 (0.01–0.81)	0.14 (0.02–1.11)	0.04 (0.03–0.30)	
ICC (%)	2.9%	5.6%	0.6%	
Health facility				
Variance	0.19 (0.10–0.33)	0.19 (0.11–0.35)	0.07 (0.04–0.13)	
ICC (%)	17.5%	19.1%	12.8%	
(C) Model fit				
AIC	5086	5014	4039	
Log Likelihood	−2540	−2503	−1990	
*P* value	–	<0.001	<0.001	

AIRR: Adjusted Incidence Rate Ratio; APD: Antepartum depression; AIC: Akaike's information criterion; ICC: Intraclass correlation coefficient.

a Adjusted for educational status, pregnancy intention of current pregnancy, number of weeks between childbirth and postpartum survey, ownership of facility of childbirth, gender of the main health worker who assisted the woman, and referral status to facility of childbirth.

After accounting for the interaction between antepartum depression scores and number of mistreatment categories experienced, and adjusting for all other variables included in Model III, we found that women's postpartum depression scores increased by 5% for each additional mistreatment category experienced (AIRR = 1.05, 95% CI: 1.01–1.09). As shown in Supplementary File S3, the analysis examining the association between specific categories of mistreatment and postpartum depression indicated that women who experienced verbal abuse had 43% higher postpartum depression scores (AIRR = 1.43; 95% CI: 1.23–1.66), while those who experienced detention in a health facility had 46% higher scores (AIRR = 1.46; 95% CI: 1.14–1.87). As shown in Supplementary File S2, the subgroup analysis of Model III stratified by women's depression status during pregnancy indicated that, among women without symptoms suggestive of antepartum depression (EPDS <11), postpartum depression score increased by 11% for each additional mistreatment category experienced (AIRR = 1.11, 95% CI: 1.06–1.17).

## Discussion

We conducted a longitudinal survey of women from the third trimester of pregnancy to up to 16 weeks postpartum to examine the association between mistreatment during facility-based childbirth and postpartum depression in Ethiopia and Guinea. Notably, our study is one of the few to have controlled for antenatal depression scores when examining the association between mistreatment and postpartum depression. Mistreatment during facility-based childbirth was widespread, with 87.4% of women in Ethiopia and 71.2% in Guinea reporting at least one of the seven categories of mistreatment. Additionally, 20.9% of participants in Ethiopia and 31.0% in Guinea had symptoms suggestive of postpartum depression. We found that for each additional category of mistreatment experienced, women's postpartum depression score increased by 5%. Among women without symptoms suggestive of antepartum depression (EPDS <11), this effect more than doubled to 11%. These findings highlight a double burden: mistreatment during facility-based childbirth not only undermines women's rights to respectful care but also significantly increases their risk of postpartum depression.

The association between mistreatment and postpartum depression observed in our study supports findings from a multi-country study conducted in urban areas in Ghana, Guinea, Myanmar and Nigeria, which reported higher odds of postpartum depression among women who had experienced verbal abuse (AOR: 1.42; 95% CI: 1.18–1.69), physical abuse (AOR: 1.57; 95% CI: 1.19–2.06), and stigma and discrimination (AOR: 1.69; 95% CI: 1.03–2.78).^17^ However, comparison between the two studies should be made with caution as the multi-country study used the Patient Health Questionnaire (PHQ-9) to assess depression, whereas our study used the EPDS. While both studies relied on postpartum surveys to assess mistreatment and postpartum depression, the effect sizes reported by the multi-country study could have been over- or underestimated as women's antepartum depressive symptoms was not included in the regression model.

A longitudinal study from Brazil also supports our findings. It reported that women who experienced three or more mistreatment categories had nearly four times the odds of severe postpartum depression (EPDS ≥15) compared to those who experienced any mistreatment (AOR = 3.86; 95%CI 1.58–9.42). Among women without symptoms of antenatal depression, the odds were even higher (AOR = 6.87; 95%CI 1.32–35.74).^27^ These results align with our findings that women's depression scores increased by 11% per additional mistreatment category experienced among women who did not have symptoms suggestive of antenatal depression. This underscores the significant role mistreatment plays in the development of postpartum depression and the urgent need to promote respectful maternity care and mitigate the mistreatment of women not only to protect their rights but promote their mental health. However, we note that the effect sizes reported in the Brazilian study may have been overestimated, as key risk factors of postpartum depression included in our analysis—such as intimate partner violence during pregnancy and the postpartum period, levels of social support, and household food security—were not accounted for in their regression models.

We believe our study provides critical evidence on the association between mistreatment during facility-based childbirth and postpartum depression, addressing a growing international call for research at the intersection of these two important issues.^16^^,^^29^ Given the negative short- and long-term consequences of postpartum depression for both mothers and their children,^30^^,^^31^ more longitudinal research is needed to better understand both the independent and interactive effects of mistreatment and postpartum depression on the use of key maternal and child health services, including postnatal care, optimal infant and young child feeding, child immunisation, and family planning.

The key strength of our study is that we used a prospective longitudinal survey and accounted for the roles of depression scores during pregnancy and relational characteristics of women (experience of intimate partner violence and social support), in addition to key sociodemographic, obstetric, and service-related characteristics, in the development of postpartum depression. Our study also provides a more precise estimate of the effect of mistreatment on postpartum depression scores due to two methodological strengths. First, depression scores during pregnancy were accounted for. Secondly, continuous postpartum depression scores were used rather than dichotomising using a cut-off employed by previous studies.^17^^,^^27^^,^^32^ This methodological approach enables to capture not only the risk of developing postpartum depression, but also the role of mistreatment in the exacerbation of existing depressive symptoms. The inclusion of two diverse settings in sub-Saharan Africa (Ethiopia and Guinea) and the application of multilevel modelling to account for variations between countries and facilities within countries are also key strengths. This will contribute to future endeavours to understand the scale of the burden and intersections of mistreatment and postpartum depression and mitigate both challenges in East and West African settings.

The findings of our study should be interpreted in the light of the following limitations. This study's single-item measure of worry about feeding the family does not fully capture household food insecurity, which could have been assessed using validated scales. Given that our study was conducted in urban settings, its findings may have limited generalisability. Broader research, particularly in rural areas, is necessary to gain a more comprehensive understanding of the intersection between mistreatment and postpartum depression. In sub-Saharan Africa, mistreatment during childbirth is often normalised by both women and health workers,^33^ which may influence how women perceive and report such experiences. Exploring these dynamics in rural populations will be essential to inform context-specific interventions that promote respectful maternity care and perinatal mental health. Furthermore, disparities in cultural and interpersonal dynamics—powerful in shaping women's perceptions of mistreatment^34^—may have contributed to discrepancies in the interpretation of the questions we used to assess the various categories of mistreatment by participants from the two countries. This is reflected in the notable differences in the prevalence of mistreatment categories between participants of the two countries. We recommend that future studies triangulate clinical profiles of participants with longitudinal surveys of pregnant and postpartum women to control for other unknown confounders and provide a more precise estimate of the causal relationships between mistreatment and postpartum depression.

Despite the growing body of research on measuring mistreatment, there is limited evidence on context-appropriate interventions for respectful maternity care. Moreover, interventions that focus primarily on the interpersonal aspects of care are often short-lived in the absence of system-wide strategies that address critical dimensions such as supplies, financing, health workforce development, and infrastructure.^35^, ^36^, ^37^ Similarly, efforts to strengthen perinatal mental health interventions in sub-Saharan Africa face challenges such as chronic underinvestment in health systems, severe workforce shortage, inadequate health workforce training in screening for and managing perinatal depression, and a heavy reliance on task-shifted maternal (mental) health services.

Given the complexity of mistreatment and postpartum depression, along with their intersecting contributors, a multidimensional and systems-oriented approach is essential for developing and implementing effective interventions.^35^^,^^38^^,^^39^ More research is needed on the organisational, social and health system-related factors that lead to mistreatment by health care workers. In testing interventions, it is important to build participants' trust and use participatory research and implementation strategies that foster meaningful engagement and avoid vilifying health workers. Such approaches enhance the credibility and impact of implementation research and innovations aimed at promoting respectful maternity care and perinatal mental health.^15^^,^^40^ Integrating respectful maternity care into perinatal mental health programme implementation (and vice versa) offers a valuable opportunity to address both mistreatment and perinatal depression, improving efficiency in the process. We draw readers’ attention to our recent viewpoint, which provides a more in-depth reflection on these critical considerations.^41^

In conclusion, we found a high prevalence of mistreatment—more than 8 in 10 women in Ethiopia and 7 in 10 in Guinea reported experiencing at least one form of mistreatment during childbirth. Additionally, 31.0% of women in Guinea and 20.9% women in Ethiopia had symptoms suggestive of postpartum depression. Each additional mistreatment category was associated with a 6% increase in women's postpartum depression score, with the effect more than twofold (13%) among women without symptoms suggestive of antenatal depression. These findings underscore the significant role mistreatment may play in the development of postpartum depression and highlight the urgent need to address mistreatment of women and promote perinatal mental health. We stress that coordinated action by stakeholders is essential to develop and implement evidence-informed and context-appropriate policies and strategies that centre women and communities, and uphold their rights and mental well-being.

## Contributors

Conceptualisation: AA; Methodology: AA, LB, BM, AD, SG; Investigation: AA, TMM, AD, SG; Formal analysis: AA, LB, TS, SG; Writing-original draft: AA; Writing-review and editing: LB, BM, CH, TMM, MA, ÖT, TM, AD, SG; Visualisation: AA; Supervision: AA, LB, BM, TMM, AD, SG—these authors had full access to and verified the underlying data; Project administration: AA, LB, BM, TMM, AD, SG; and funding acquisition: AA, LB, BM, TMM, AD, SG. All authors read and approved the final version of the manuscript.

## Data sharing statement

Request to access the dataset used for this study can be sent to the principal investigator (AA). The principal investigator will then forward the request to the Data Access Committee at the Institute of Tropical Medicine which reviews requests case-by-case and make decisions as per the committee's guidelines.

## Declaration of interest

All authors declare that they have no conflicts of interest related to this work.
