# Supplementary material for: Unraveling the link between the mistreatment of women during childbirth and postpartum depression: a prospective longitudinal study in Ethiopia and Guinea
Source: eClinicalMedicine. 2025 Dec 18;91:103702. doi: 10.1016/j.eclinm.2025.103702 (PMC12774692; doi:10.1016/j.eclinm.2025.103702)
Supplement: Supplementary File 1 [file mmc1.pdf]

Supplementary file 1: Questionnaires
Supplementary file 1.A
Questionnaire for women's survey during pregnancy

Name of data collector:___________________________________

Screening questions

Have you been living in Addis Ababa/Conakry over the last six months?

Yes ☐ No ☐ *If "No", thank the participant and stop the survey.*

What is your current gestational age (in weeks)? _______weeks. *If "<28 weeks", thank the participant and stop the survey*

Health Facility: ________________________

Four-digit questionnaire code: ___________

Date of data collection: ______________

Time of data collection (start): ______________

Section I: Socio-demographic and household characteristics

S.No	Questions	Possible responses	Remark/skip
101.	In which sub-city/commune do you live?	________________	
102.	How long have you lived in Addis Ababa/Conakry?<br>*Note: Round off using 6 months as a cut-off point. Example:1 year and 6 months should be written as 2 years*	________years	
103.	What is your current age/how old are you today (in completed years)?	A. _______________ years<br>B. Don't know/refused to answer	
104.	How old were you at your first pregnancy (in completed years)?	A. _______________ years<br>B. Don't know/refused to answer	
105.	What is your current marital status?	1. Single<br>2. Married<br>3. Cohabiting<br>4. Separated<br>5. Divorced<br>6. Widowed<br>7. Don't know / refused to answer	
106.	What is your highest educational status?	1. No education<br>2. Primary - not completed grade 8<br>3. Completed grade 8<br>4. Secondary - not completed grade 12<br>5. Completed grade 12<br>6. More than secondary<br>7. Don't know / refused to answer	
107.	What is your current occupation?	1. Housewife<br>2. Farmer<br>3. Private Employee<br>4. Government Employee<br>5. Private business<br>6. Others: specify...................<br>7. Don't know / refused to answer	
108.	How many alive child/children do you have?	A. _______________child/children<br>B. Don't know / refused to answer	

109.	How many people (including adults and children, and including you) live in your household?	A. ________________persons<br>B. Don't know / refused to answer	
110.	Do you have regular household monthly cash income?	1. Yes<br>2. No<br>3. Don't know / refused to answer	**If 2/3 → 114**
111.	What is your household's monthly income (in Birr)?	A. ________________ Eth. Birr<br>B. Don't know / refused to answer	
112.	In the past four weeks, how often have you worried that you cannot feed your family?	1. Not at all<br>2. Hardly ever<br>3. Sometimes<br>4. Very often<br>5. Don't know / refused to answer	

Section II: Obstetric and previous service utilisation characteristics

S.No	Questions	Possible responses	Remark/skip
201.	How many times have you given birth before?	_____________________ times	**If 0 → 204**
202.	Where did you give birth your last (previous) child?	1. At health facility<br>2. At home<br>3. At traditional birth attendant's home<br>4. On the way to health facility<br>5. Others: specify...................<br>6. Don't know / refused to answer	
203.	How many times have you given birth in a health facility?	A. _____________________ times<br>B. Don't know / refused to answer	
204.	Was your current pregnancy planned at the time you got pregnant?	1. Yes, I wanted a child now<br>2. No, I wanted a child later<br>3. No, I did not want a child at all	
205.	Are you pregnant with one baby or twins?	1. Singleton<br>2. Multiple (two or more)<br>3. I don't know	
206.	How many antenatal care visits did you have during your current pregnancy, including your visit today?	A. _________ visit/s<br>B. Don't know / refused to answer	
207.	Have you had any complications during your current pregnancy?	1. Yes, specify _____________<br>2. No<br>3. Don't know / refused to answer	

Section III: Social support and marital satisfaction

Read this for the woman: "the following statements are related to social support you receive from your husband/partner/families /relatives/friends. I will read the statements one by one; you have five options to choose from".

S.No	Questions	Possible responses					
For each of the following statements, please circle one response which shows how the woman feels about the support she has right now		**Always**	**Most of the time**	**Some of the time**	**Rarely**	**Never**	**Don't know or refused**
301.	I have good friends who support me	5	4	3	2	1	99
302.	My family is always there for me	5	4	3	2	1	99

303.	My husband/partner helps me a lot<br>***Note: skip if the woman is NOT married or cohabited***	5	4	3	2	1	99
304.	There is conflict with my husband/partner<br>***Note: skip if the woman is NOT married or cohabited***	1	2	3	4	5	99
305.	I feel controlled by my husband/partner<br>***Note: skip if the woman is NOT married or cohabited***	1	2	3	4	5	99
306.	I feel loved by my husband/partner<br>***Note: skip if the woman is NOT married or cohabited***	5	4	3	2	1	99

Section IV: Respectful antenatal care

Read this for the woman: the following questions are related to your antenatal care experience in your current visit. I will read the questions one by one; you have three options to choose from: "Yes" or "No" or "NA (Not applicable)". Dear data collector, mark only on the corresponding response

S.No	Questions	Possible responses			Remark/skip
During your current antenatal check:		**Yes**	**No**	**NA/refused**	
401.	Did the health workers use harsh or rude language?	1	0	3	
402.	Did the health workers make judgmental or accusatory comments about you?	1	0	3	
403.	Were you beaten, slapped, kicked, or pinched?	1	0	3	
404.	Were you gagged?	1	0	3	
405.	Were you physically restrained?	1	0	3	
406.	Did the health workers make threats of withholding treatment?	1	0	3	
407.	Did the health worker/s blame you for being pregnant or for any aspects of your pregnancy?	1	0	3	
408.	Did the health workers obtain your consent for all procedures, including physical examination?	1	0	3	
409.	Did the health workers keep information about you confidential?	1	0	3	
410.	Were you left in the antenatal care clinic for a prolonged period of time without attention?	1	0	3	
411.	Did the health workers conduct an abdominal examination for you?				**If 0/3 →414**
412.	Did the health workers ask your permission before conducting an abdominal examination?	1	0	3	
413.	Did the health workers conduct abdominal examination without maintaining your privacy?	1	0	3	
414.	Did the health workers speak to you in a language you do not understand?	1	0	3	
415.	Did you want to have a companion in the antenatal clinic?	1	0	3	**If 0/3 →417**
416.	Did the health workers allow you to have your companion present?	1	0	3	
417.	Did the health workers make you stay in the health facility against your will?	1	0	3	

S.No	Questions	Possible responses			Remark/skip
418.	Did the health workers discriminate against you based on your religion /ethnicity/age/socioeconomic status/medical condition?	1	0	3	
419.	Do you think you have been mistreated (disrespected or abused) by a health worker during your antenatal visit today?	1. Yes<br>2. No<br>3. Don't know or refused to answer			

Section V: Antepartum depression screening

Read this for the woman: the following statements are related to your experience of mental health in the past 7 days. I will read the statements one by one; you have four options to choose from.

Dear data collector, circle only on the corresponding response the woman made

S.No	Questions	Possible responses	Remark
In the past 7 days			
501.	I have been able to laugh and see the funny side of things	1. As much as I always could<br>2. Not quite so much now<br>3. Definitely not so much now<br>4. Not at all	
502.	I have looked forward with enjoyment to things	1. As much as I ever did<br>2. Rather less than I used to<br>3. Definitely less than I used to<br>4. Hardly at all	
503.	I have blamed myself unnecessarily when things went wrong	1. Yes, most of the time<br>2. Yes, some of the time<br>3. Not very often<br>4. No, never	
504.	I have been anxious or worried for no good reason	1. No, not at all<br>2. Hardly ever<br>3. Yes, sometimes<br>4. Yes, very often	
505.	I have felt scared or panicky for no very good reason	1. Yes, quite a lot<br>2. Yes, sometimes<br>3. No, not much<br>4. No, not at all	
506.	Things have been getting on top of me	1. Yes, most of the time I haven't been able to cope at all<br>2. Yes, sometimes I haven't been coping as well as usual<br>3. No, most of the time I have coped quite well<br>4. No, I have been coping as well as ever	
507.	I have been so unhappy that I have had difficulty sleeping	1. Yes, most of the time<br>2. Yes, sometimes<br>3. Not very often<br>4. No, not at all	
508.	I have felt sad or miserable	1. Yes, most of the time<br>2. Yes, sometimes<br>3. Not very often<br>4. No, not at all	

S.No	Questions	Possible responses	Remark
509.	I have been so unhappy that I have been crying	1. Yes, most of the time<br>2. Yes, quite often<br>3. Only occasionally<br>4. No, never	
510.	The thought of harming myself has occurred to me	1. Yes, quite often<br>2. Sometimes<br>3. Hardly ever<br>4. Never	
511.	Did you have any history of depression before your current/index pregnancy?	1. Yes<br>2. No<br>3. I don't know	

Section VI: Spousal violence

Screening question

How long have you been married or cohabited – only current union (months)? ______ months

Note: skip this section for women who were not married or cohabited in the 12 months preceding the survey

Enumerator: ensure auditory privacy!

Read this for the woman: the following statements are related to your experience of violence by your partner/husband in the past 12 months. I will read the statements one by one; you have three options to choose from "Yes" or "No" or "I don't know".

S.No	Questions	Possible responses			Remark/skip
In the last 12 months, did your last husband/partner ever:		**Yes**	**No**	**I don't know**	
601.	Push you, shake you, or throw something at you?	1	0	3	
602.	Slap you or twist your arm?	1	0	3	
603.	Punch you with his fist or with something that could hurt you?	1	0	3	
604.	Kick you or drag you?	1	0	3	
605.	Try to strangle you or burn you?	1	0	3	
606.	Threaten you with a knife, gun, or other type of weapon?	1	0	3	
607.	Attack you with a knife, gun, or other type of weapon?	1	0	3	
608.	Physically force you to have sexual intercourse with him even when you did not want to?	1	0	3	
609.	Force you to perform other sexual acts you did not want to?	1	0	3	
610.	Said or did something to humiliate you in front of others?	1	0	3	
611.	Threatened to hurt or harm you or someone you cared about?	1	0	3	
612.	Insulted you or made you feel bad about yourself?	1	0	3	

Thank you for your participation!

Time (end of survey): ______________

Supplementary file 1.B

Questionnaire for women's survey during the postpartum period

Sub-city/commune (place of data collection): ________________________

Four-digit questionnaire code: ___________

Date of data collection: ______________

Time of data collection (start): ______________

Place of data collection: At woman's house ☐ Health facility ☐ Other (specify) _____________

Section I: Socio-demographic and household characteristics (update from round 1)

Can I confirm that some information we have about you is still correct?

S.No	Questions	Possible responses	Remark/skip
101	Current marital Status	1. Single<br>2. Married<br>3. Cohabiting<br>4. Separated<br>5. Divorced<br>6. Widowed<br>7. Don't know / refused to answer	
102	In the past four weeks, how often have you worried that you cannot feed your family?<br>***Please read the options***	1. Not at all<br>2. Hardly ever<br>3. Sometimes<br>4. Very often<br>5. Don't know / refused to answer	

Section II: Social support and marital satisfaction

Read this for the woman: "the following statements are related to social support you receive from your husband/partner/families /relatives/friends. I will read the statements one by one; you have five options to choose from".

S.No	Questions	Possible responses					
For each of the following statements, please circle one response which shows how the woman feels about the support she has right now		**Always**	**Most of the time**	**Some of the time**	**Rarely**	**Never**	**Don't know or refused**
201.	I have good friends who support me	5	4	3	2	1	99
202.	My family is always there for me	5	4	3	2	1	99
203.	My husband/partner helps me a lot<br>***Note: skip if the woman is NOT married or cohabited***	5	4	3	2	1	99
204.	There is conflict with my husband/partner<br>***Note: skip if the woman is NOT married or cohabited***	1	2	3	4	5	99
205.	I feel controlled by my husband/partner<br>***Note: skip if the woman is NOT married or cohabited***	1	2	3	4	5	99
206.	I feel loved by my husband/partner<br>***Note: skip if the woman is NOT married or cohabited***	5	4	3	2	1	99

Section III: current childbirth related characteristics

S.No	Questions	Possible responses	Remark/skip
301.	When did you give birth?	___*dd*___*mm*___*yy*	

S.No	Questions	Possible responses	Remark/skip
302.	What was the outcome of the participant's index pregnancy? *(do not ask this question; rather complete based on what you see or the participant's responses to other questions/initial phone call)*	1. Live birth<br>2. Live birth but infant death afterward, specify age at death _______days<br>3. Stillbirth	
303.	Did you have any complication during your index pregnancy?	1. Yes, specify _____________<br>2. No<br>3. Don't know / refused to answer	
304.	Did you have any complication during your childbirth?	1. Yes, specify _______________<br>2. No<br>3. Don't know / refused to answer	
305.	Where did you give birth?	1. At a health facility<br>2. On the way to health facility<br>3. At home and went to health facility thereafter<br>4. At home<br>5. At traditional birth attendant's home/other home setting	**If 4/5 → 319**
306.	Facility name	______________________<br>____________________	
307.	Do you know what kind of facility this is?	1. Public health centre<br>2. Public general/referral hospital<br>3. Public specialised teaching hospital<br>4. Private hospital<br>5. Private higher clinic<br>6. Private not-for-profit<br>7. Don't know	
308.	Were you referred to this facility from another facility or directly went to the facility for childbirth?	1. Referred from another facility<br>2. Non-referred (direct visit)	**If 2 → 310**
309.	How many health facilities did you visit as part of the referral process?	____________________	
310.	When did you get admitted to the health facility you gave birth at or you visited after giving birth at home?	_______AM *(___dd___mm___yy)*<br>_______PM *(___dd___mm___yy)*	
311.	What time did you give birth?	_______AM *(___dd___mm___yy)*<br>_______PM *(___dd___mm___yy)*	
312.	What type of ward were you in?	1. Private ward<br>2. Shared ward<br>3. Don't know / refused to answer	
313.	What was the gender of the service provider who mainly assisted you during your labour and childbirth?	1. Female<br>2. Male<br>3. Don't know	
314.	What type of birth did you have?	1. Vaginal birth<br>2. Assisted vaginal birth<br>3. Caesarean birth after labour trial<br>4. Caesarean birth without labour trial<br>5. Don't know	
315.	Did you have any procedure for an assisted delivery?	1. Yes<br>2. No	**If 2/3 → 317**

S.No	Questions	Possible responses	Remark/skip
		3. DK/refused	
316.	Which procedure did you receive? ***(Multiple responses possible)***	**Yes** / **No**<br>Vacuum: 1 / 2<br>Forceps: 1 / 2<br>Episiotomy (not tear): 1 / 2	
317.	How long did you stay at the health facility?	A. _____ number of hours<br>B. _____ number of days<br>C. _____ number of weeks<br>D. don't know or refused to answer	
318.	Did you have a postnatal check by a health professional before you were discharged from the health facility after your childbirth?<br>***NB: skip if the woman gave birth at home***	1. Yes<br>2. No	
319.	Did you have a postnatal check by a health professional after your childbirth?<br>*(NB: postnatal check after discharge if birth happened at a health facility)*	1. Yes, at home<br>2. Yes, at a health facility<br>3. Yes, both at home and a health facility<br>4. No<br>5. Don't know or refused to answer	
320.	Did you intend to breastfeed your baby?<br>***NB: skip if the woman's response to Q302 is 2/3***	1. Yes<br>2. No	***If 2/3 for Q302*** **→ 401**
321.	Did you breastfeed yesterday sunrise to today sunrise?	1. Yes<br>2. No<br>3. Don't know or refused to answer	
322.	Did you have to stop breastfeeding due to circumstances beyond your control?	1. Yes<br>2. No<br>3. Don't know or refused to answer	
323.	How happy are you with your breastfeeding practice?<br>*Please read the options*	1. Very happy<br>2. Happy<br>3. Neutral<br>4. Unhappy<br>5. Very unhappy<br>6. Don't know or refused to answer	
324.	Do you have a card or another document where your newborn's vaccinations are written down?	1. Yes, card or another document seen<br>2. No, no card or other document seen	**If 2 → 327**
325.	*Data collector: copy the following information from the vaccination card or document*	Yes / No<br>BCG: ___ / ___<br>Polio-0: ___ / ___<br>Polio-1: ___ / ___<br>Penta-1: ___ / ___	
326.	I want to take the picture of the newborn's vaccination card or document so that it will help me to counter check the information I filled in. Are you willing?	1. Yes<br>2. No	

S.No	Questions	Possible responses	Remark/skip
	Data collector: if the caregiver consented take the snapshot and upload the picture.		
327.	Has the newborn received a BCG vaccination against tuberculosis, that is, an injection in the arm or shoulder that usually causes a scar?	1. Yes<br>2. No<br>3. I don't know/refused to answer	
328.	Has the newborn received oral polio vaccine, that is, two drops in the mouth to prevent polio?	1. Yes<br>2. No<br>3. I don't know/refused to answer	**If 2/3 → 331**
329.	Did the newborn receive the first oral polio vaccine in the first two weeks after birth or later?	1. In the first two weeks<br>2. Later	
330.	How many times did the newborn receive the oral polio vaccine?	__________ times	
331.	Has the newborn ever received a pentavalent vaccination, that is, an injection usually given on the left upper thigh sometimes at the same time as polio drops?	1. Yes<br>2. No<br>3. I don't know/refused to answer	

Section IV: Mistreatment assessment questions

Read this for the woman: the following questions are related to your experience in your current childbirth. I will read the questions one by one; you have three options to choose from: "Yes" or "No" or "DK (I do not know)". Dear data collector, select only one corresponding response.

!! Skip this section if response to Q305 is 4/5

S.No	Question	Possible responses			Remark/skip
During this admission for childbirth…		**Yes**	**No**	**DK**	
401.	Did the health workers use harsh or rude language?	1	0	3	
402.	Did the health workers make judgmental or accusatory comments about you?	1	0	3	
403.	Were you beaten, slapped, kicked, or pinched during childbirth?	1	0	3	
404.	Were you gagged during childbirth?	1	0	3	
405.	Were you physically restrained during childbirth?	1	0	3	
406.	Did the health workers make threats of withholding treatment?	1	0	3	
407.	Did the health workers blame you for any feature of your birth outcomes?	1	0	3	
408.	Did the health workers obtain your consent for all procedures?	1	0	3	
409.	Did the health workers keep information about you confidential?	1	0	3	
410.	Did you have any surgical procedure (episiotomy, caesarean section)?	1	0	3	**If 0/3 → 412**
411.	Did the provider ask your permission before performing the surgical procedure?	1	0	3	
412.	Did the health workers always come following your call?	1	0	3	

S.No	Question	Possible responses			Remark/skip
413.	Were you ever left for a prolonged period of time without attention during your labour or postpartum care?	1	0	3	
414.	Was a health provider present for the actual birth of your baby?	1	0	3	**Skip if 2/3/4/5 for Q 305**
415.	Did the health workers ever separate you from your baby without explaining the reason?	1	0	3	
416.	Did the health workers ask your permission before conducting a vaginal examination?	1	0	3	
417.	Did any provider conduct vaginal examination without maintaining your privacy?	1	0	3	
418.	Did the health workers speak to you in a language you do not understand?	1	0	3	
419.	Did the health workers give you periodic updates on your labor?	1	0	3	**Skip if 2/3/4/5 for Q 305**
420.	Did you want to have a birth companion during your labour and childbirth in the labor ward?	1	0	3	**Skip if 2/3/4/5 for Q 305<br>If 0 →422**
421.	Did the health workers allow you to have your birth companion present?	1	0	3	
422.	Did you want to move around during your labor?	1	0	3	**Skip if 2/3/4/5 for Q 305<br>If 0 →424**
423.	Did the health workers allow you to move around during your labor?	1	0	3	
424.	Did you want to have food or fluids during your labor?	1	0	3	**Skip if 2/3/4/5 for Q 305<br>If 0 →426**
425.	Did the health workers allow you to have food or fluids?	1	0	3	
426.	Did you have a preferred birthing position?	1	0	3	**Skip if 2/3/4/5 for Q 305<br>If 0 →428**
427.	Did the health workers allow you to give birth in your preferred position?	1	0	3	
428.	Did you want to have a cultural practice in labor?	1	0	3	**Skip if 2/3/4/5 for Q 305<br>If 0 →430**
429.	Did the health workers allow you this cultural practice in labor?	1	0	3	
430.	Did the health workers make you stay in the health facility against your will?	1	0	3	
431.	Did the health workers discriminate against you based on your religion /ethnicity/age/socioeconomic status/medical condition?	1	0	3	
432.	Do you think you have been mistreated by a health worker during your recent childbirth?	1	0	3	
433.	Have you been mistreated by other people outside the maternity care setting (for example guards, registration desk officers, other administrative and support staff, etc)?	1	0	3	

Section V: Postpartum depression screening questions

Read this for the woman: the following statements are related to your experience of mental health in the past 7 days. I will read the statements one by one; you have four options to choose from.

Dear data collector, circle only on the corresponding response the woman made

S.No	Questions	Possible responses	Remark
In the past 7 days			

S.No	Questions	Possible responses	Remark
501.	I have been able to laugh and see the funny side of things	1. As much as I always could<br>2. Not quite so much now<br>3. Definitely not so much now<br>4. Not at all	
502.	I have looked forward with enjoyment to things	1. As much as I ever did<br>2. Rather less than I used to<br>3. Definitely less than I used to<br>4. Hardly at all	
503.	I have blamed myself unnecessarily when things went wrong	1. Yes, most of the time<br>2. Yes, some of the time<br>3. Not very often<br>4. No, never	
504.	I have been anxious or worried for no good reason	1. No, not at all<br>2. Hardly ever<br>3. Yes, sometimes<br>4. Yes, very often	
505.	I have felt scared or panicky for no very good reason	1. Yes, quite a lot<br>2. Yes, sometimes<br>3. No, not much<br>4. No, not at all	
506.	Things have been getting on top of me	1. Yes, most of the time I haven't been able to cope at all<br>2. Yes, sometimes I haven't been coping as well as usual<br>3. No, most of the time I have coped quite well<br>4. No, I have been coping as well as ever	
507.	I have been so unhappy that I have had difficulty sleeping	1. Yes, most of the time<br>2. Yes, sometimes<br>3. Not very often<br>4. No, not at all	
508.	I have felt sad or miserable	1. Yes, most of the time<br>2. Yes, sometimes<br>3. Not very often<br>4. No, not at all	
509.	I have been so unhappy that I have been crying	1. Yes, most of the time<br>2. Yes, quite often<br>3. Only occasionally<br>4. No, never	
510.	The thought of harming myself has occurred to me	1. Yes, quite often<br>2. Sometimes<br>3. Hardly ever<br>4. Never	

Section VI: Spousal violence

Enumerator: ensure auditory privacy!

Note: only ask if the woman who was married/cohabiting since her recent birth

Read this for the woman: the following statements are related to your experience of violence by your partner/husband since your recent birth. I will read the statements one by one; you have three options to choose from.

S.No	Questions	Possible responses			Remark/skip
Since your recent birth, did your last husband/partner ever:		**Yes**	**No**	**I don't know**	
601.	Push you, shake you, or throw something at you?	1	0	3	
602.	Slap you or twist your arm?	1	0	3	
603.	Punch you with his fist or with something that could hurt you?	1	0	3	
604.	Kick you or drag you?	1	0	3	
605.	Try to strangle you or burn	1	0	3	
606.	Threaten you with a knife, gun, or other type of weapon?	1	0	3	
607.	Attack you with a knife, gun, or other type of weapon?	1	0	3	
608.	Physically force you to have sexual intercourse with him even when you did not want to?	1	0	3	
609.	Force you to perform other sexual acts you did not want to?	1	0	3	
610.	Said or did something to humiliate you in front of others?	1	0	3	
611.	Threatened to hurt or harm you or someone you cared about?	1	0	3	
612.	Insulted you or made you feel bad about yourself?	1	0	3	

Thank you for your participation!

Time (end of survey): __________
