# Supplementary material for: Unraveling the link between the mistreatment of women during childbirth and postpartum depression: a prospective longitudinal study in Ethiopia and Guinea
Source: eClinicalMedicine. 2025 Dec 18;91:103702. doi: 10.1016/j.eclinm.2025.103702 (PMC12774692; doi:10.1016/j.eclinm.2025.103702)
Supplement: Supplementary File 2 [file mmc2.pdf]

Supplementary file 2

A) Multilevel mixed-effects model assessing the association between mistreatment and postpartum depression scores among women without symptoms suggestive of antepartum depression (EPDS < 11)

Variables	AIRR (95% CI)	p value
**Fixed effects**		
(Intercept)	2.58 (1.30–5.13)	0.007
Number of mistreatment categories experienced	1.11 (1.06–1.17)	< 0.001
Antepartum depression score	1.11 (1.07–1.14)	< 0.001
Number of mistreatment categories experienced * APD score	0.99 (0.97–0.99)	0.005
Age (in years) at the time of the survey during pregnancy	1.02 (1.01–1.04)	0.003
Parity during pregnancy		
Nullipara (no previous births)	Ref.	
Primipara (1 previous birth)	0.92 (0.80–1.07)	0.300
Multipara (2 or more previous births)	0.85 (0.71–1.02)	0.074
Complication(s) during pregnancy		
No	Ref.	
Yes	0.88 (0.75–1.03)	0.099
Experienced any form of IPV in the 12 months preceding the survey during pregnancy		
No	Ref.	
Yes	1.04 (0.93–1.17)	0.512
Social support score during the postpartum survey	0.95 (0.94–0.98)	< 0.001
Pregnancy outcome		
Live birth	Ref.	
Stillbirth or early neonatal loss	2.18 (1.67–2.84)	< 0.001
Had postnatal checkup after childbirth		
No	Ref.	
Yes	0.92 (0.82–1.04)	0.198
Worried about feeding family in the two weeks preceding the postpartum survey		
No	Ref.	
Yes	1.91 (1.70–2.14)	< 0.001
Mode of childbirth		
Vaginal	Ref.	
Caesarean	1.20 (0.96–1.50)	0.105
Had procedure for assisted vaginal delivery		
No	Ref.	
Yes	1.23 (1.08–1.42)	0.002
Complication/s during childbirth		
No	Ref.	
Yes	1.02 (0.88–1.19)	0.755
Experienced any form of IPV between childbirth and postpartum survey		
No	Ref.	
Yes	1.04 (0.91–1.19)	0.577

**Adjusted for educational status, pregnancy intention of current pregnancy, number of weeks between childbirth and postpartum survey, ownership of facility of childbirth, and referral status to facility of childbirth*

AIRR: Adjusted Incidence Rate Ratio; APD: Antepartum depression

B) Multilevel mixed-effects model assessing the association between mistreatment and postpartum depression scores among women with symptoms suggestive of antepartum depression (EPDS ≥ 11)

Variables	AIRR (95% CI)	p value
**Fixed effects**		
(Intercept)	11.41 (4.12–31.65)	< 0.001
Number of mistreatment categories experienced	0.98 (0.88–1.10)	0.934
Antepartum depression score	1.00 (0.97–1.05)	0.679
Number of mistreatment categories experienced * APD score	1.00 (0.99–1.01)	0.751
Age (in years) at the time of the survey during pregnancy	1.01 (0.99–1.03)	0.076
Parity during pregnancy		
Nullipara (no previous births)	Ref.	
Primipara (1 previous birth)	0.69 (0.53–0.98)	0.007
Multipara (2 or more previous births)	0.79 (0.60–1.03)	0.086
Complication(s) during pregnancy		
No	Ref.	
Yes	1.25 (1.01–1.53)	0.037
Experienced any form of IPV in the 12 months preceding the survey during pregnancy		
No	Ref.	
Yes	0.87 (0.73–1.02)	0.093
Social support score during the postpartum survey	0.98 (0.95–1.00)	0.070
Pregnancy outcome		
Live birth	Ref.	
Stillbirth or early neonatal loss	0.77 (0.54–1.09)	0.143
Had postnatal checkup after childbirth		
No	Ref.	
Yes	1.06 (0.90–1.25)	0.482
Worried about feeding family in the two weeks preceding the postpartum survey		
No	Ref.	
Yes	1.35 (1.15–1.58)	< 0.001
Mode of childbirth		
Vaginal	Ref.	
Caesarean	0.92 (0.61–1.38)	0.673
Had procedure for assisted vaginal delivery		
No	Ref.	
Yes	1.10 (0.87–1.39)	0.439
Complication/s during childbirth		
No	Ref.	
Yes	1.26 (1.03–1.55)	0.024
Experienced any form of IPV between childbirth and postpartum survey		
No	Ref.	
Yes	1.29 (1.05–1.59)	0.015

**Adjusted for educational status, pregnancy intention of current pregnancy, number of weeks between childbirth and postpartum survey, ownership of facility of childbirth, and referral status to facility of childbirth*

AIRR: Adjusted Incidence Rate Ratio; APD: Antepartum depression
