# Supplementary material for: Unraveling the link between the mistreatment of women during childbirth and postpartum depression: a prospective longitudinal study in Ethiopia and Guinea
Source: eClinicalMedicine. 2025 Dec 18;91:103702. doi: 10.1016/j.eclinm.2025.103702 (PMC12774692; doi:10.1016/j.eclinm.2025.103702)
Supplement: Supplementary File 3 [file mmc3.pdf]

Supplementary File 3: Multilevel mixed-effects model assessing the association between specific categories of mistreatment and postpartum depression scores

Mistreatment category*	AIRR (95% CI)	p value
Lack of information, privacy, and confidentiality	1.01 (0.89-1.14)	0.936
Non-consented care	1.04 (0.92-1.18)	0.507
Refusal of preference	0.94 (0.82-1.08)	0.397
Neglect and discrimination	1.14 (1.00-1.29)	0.056
Physical abuse	1.04 (0.89-1.21)	0.606
Verbal abuse	1.43 (1.23-1.66)	<0.001
Detention in health facility	1.46 (1.14-1.87)	0.003

**For each category: adjusted for educational status, pregnancy intention of current pregnancy, number of weeks between childbirth and postpartum survey, ownership of facility of childbirth, referral status to facility of childbirth, parity, complications during pregnancy, experience of IPV in the 12 months preceding the survey during pregnancy, social support score, pregnancy outcome, postnatal checkup, worry about feeding family, mode of childbirth, procedure for assisted vaginal delivery, complications during childbirth, and experience of IPV after childbirth*

AIRR: Adjusted Incidence Rate Ratio; APD: Antepartum depression
